# A cross-sectional study on caregivers’ perspective of the quality of life and adherence of paediatric HIV patients to highly active antiretroviral therapy

**DOI:** 10.1186/s12887-020-02194-7

**Published:** 2020-06-09

**Authors:** Michael Lahai, Peter Bai James, Noel Nen’man Wannang, Haja Ramatulai Wurie, Sorie Conteh, Abdulai Jawo Bah, Mohamed Samai

**Affiliations:** 1 Faculty of Pharmaceutical Sciences, College of Medicine and Allied Health Sciences, University of Sierra Leone, Freetown, 00232 Sierra Leone; 2 Department of Pharmacology and Toxicology, University of Jos, Jos, Nigeria; 3 Faculty of Basic Medical Sciences, College of Medicine and Allied Health Sciences, University of Sierra Leone, Freetown, Sierra Leone; 4 Faculty of Clinical Sciences, College of Medicine and Allied Health Sciences, University of Sierra Leone, Freetown, Sierra Leone

**Keywords:** Awareness, stigma, Disclosure, Caregiver, Nuclear family, Discrimination

## Abstract

**Background:**

Poor compliance to highly active antiretroviral therapy (HAART) can result in the poor quality of life in children living with Human immunodeficiency virus/Acquired immunodeficiency syndrome (HIV/AIDS) because of low plasma drug concentration and the possibility of drug resistance. This study evaluates the response of caregivers for determination of adherence and the four quality of life domains in children (aged 14 years and under) on HAART.

**Methods:**

We conducted a cross-sectional study of 188 children, each accompanied by their caregivers at Ola During Children’s Hospital and Makeni Government Hospital between September and November 2016. Adherence to HAART and Quality of life was assessed using the WHO Quality of life summary questionnaire (WHOQOL-BREF). We obtained ethical approval from the Sierra Leone Ethics and Scientific Review Committee.

**Results:**

The study revealed 5.9% adherence amongst paediatric patients, and a strong association of adherent patients(*p* = 0.019*) to the physical health domain (mean = 64.61 SD = 8.1). Caregiver HIV status showed a strong association with the physical (mean = 58.3, SD = 11.7 and *p* = 0.024*), and psychological health domains (mean = 68.2, SD = 14.7 and *p* = 0.001). Caregiver type (mother/father/sibling) accompanying child to hospital also showed strong associated with the physical (mean = 58.0, SD = 10.6, *p* <  0.001), psychological (mean 68.2 SD = 14.81 *p* <  0.001) and environmental health domains (mean = 59.7, SD = 13.47, p <  0.001). Further regression analysis showed a strong association with physical health domain for HIV positive caregivers (*p* = 0.014) and adherent paediatric patients (*p* = 0.005). Nuclear family also showed a strong association with psychological (*p* <  0.001) and environmental (*p* = 0.001) health domains.

**Conclusion:**

This study showed a strong association between the quality of life domains and the involvement of nuclear family caregiver, HIV-positive caregiver and adherence to HAART. Our study suggests that the involvement of any member of the nuclear family, HIV positive parents and patient adherence to therapy can improve the quality of life of paediatric HIV/AIDS patients on highly active antiretroviral therapy in the two hospitals.

## Background

Children and adolescents make up 33.3% of the world’s population [1]. In 2014, two million six hundred children aged 0 to 15 years were known to be living with HIV/AIDS globally with only a third of them accessing antiretroviral treatment. Human immunodeficiency virus/Acquired immunodeficiency syndrome (HIV/AIDS) is the second most significant cause of death among adolescents and a leading cause of death in Africa among adolescents, most of whom got HIV as infants [2]. In 2016, United Nations Joint Program on HIV/AIDS (UNAIDS) estimated that 28% of children aged 14 years and under are living with HIV/AIDS in Western and Central Africa, with 40% of these children dying from AIDS-related illnesses [3].

Studies have shown that non-adherence to highly active antiretroviral therapy (HAART) is associated with poor quality of life [4]. In contrast, the provision of appropriate HIV care can lead to an improvement in health-related quality of life among people living with HIV/AIDS [5]. Similarly, studies using clinical and immunological markers have shown that early introduction of highly active antiretroviral therapy in children with HIV/AIDS can have a positive influence on their quality of life [6]. Good clinical outcomes have also been observed as a direct effect of HAART adherence with a known reduction in morbidity and mortality in children infected with HIV in Kenya [7]. Non-adherence is defined as the discontinuation of part or all of the treatment regimen that includes missing dose, under-dose, over-dose and drug holidays [8]. The key drivers of non-adherence include lack of insight, forgetfulness, busy work schedule, distance to clinic and medication beliefs, and it has been shown that African states do share similar drivers of non-adherence with western nations [9]. The International Association of Physicians in AIDS Care recommends routine monitoring of adherence to evaluate adherence interventions and prevent drug resistance. Studies have also shown that achievement of good quality of life requires a high level of adherence of over 95% for paediatric patients on HAART for whom there is also no specific recommendation for monitoring adherence [9]. It is known that caregiver estimates for HAART adherence in children are consistently higher than adherence by other measures such as pharmacy refill and other new technologies, suggesting that caregivers may overestimate their child’s adherence. However, outside funded research settings, new technologies such as Medication Event Monitoring Systems are usually too expensive than caregiver estimates [10]. Therefore, despite the limitation associated with caregiver estimates of adherence, it remains the most widely used method of adherence in most low and middle-income countries [11].

HIV prevalence in Sierra Leone is 1.5%, and prevalence among children is 5.8% [12]. Sierra Leone also has 37.7% antiretroviral therapy coverage among all age groups, with an estimated 383 children receiving antiretroviral therapy (ART) and 1810 children in need of antiretroviral therapy [13].

The 2014 Ebola epidemic resulted in a reduction in access to HIV/AIDS care because most parents were unwilling to seek care at hospitals due to Ebola-related stigma and the fear associated with becoming infected with the Ebola virus as well as mistrust for healthcare workers [14]. The end of the Ebola outbreak saw the implementation of post-Ebola interventions aimed at improving healthcare service utilisation among people living with HIV/AIDS (PLHIV). These interventions include identification of loss to treatment follow-up and public awareness [13]. Most HIV/AIDS studies in Sierra Leone are focused on knowledge, attitudes and behaviour of high-risk groups like sex workers and youths [15–17]. Currently, there is little or no research evidence on the level of adherence and the quality of life of children living with HIV/AIDS (CLHIV) in Sierra Leone.

This study adds to the contemporary HIV/AIDS literature in Sierra Leone and in Africa, by assessing adherence to HAART among paediatric HIV/AIDS patients through the perception of their caregivers. Our study also sought to determine the association between the demographic and health-related factors of caregivers and the quality of life of paediatric HIV/AIDS patients in two public hospitals in Sierra Leone.

## Methods

### Study design, setting and population

We conducted cross-sectional study of caregivers accompanying HIV-infected children aged 14 years and under, between 1st September and 30th November 2016. A caregiver was defined in our study as a parent or guardian accompanying HIV-infected children aged 14 years and below.

We conducted our study at the HIV/AIDS clinics of Ola During Children’s Hospital (ODCH) and Makeni Government Hospital located in the Western and Northern regions of Sierra Leone respectively. These hospitals are the main referral hospitals in two of Sierra Leone’s four regions. A convenient sampling method was used to recruit caregivers accompanying HIV-infected children in our study. All caregivers accompanying HIV-infected children who seek care at these hospitals between the 1st September to 30th November 2016 were invited to take part in the study. At the end of November 2016, 200 caregivers accompanying HIV-infected children were invited to participate in the study. However, only one hundred and two caregivers from Ola During Children’s Hospital and 86 caregivers from Makeni Government Hospital consented to participate in this study. Nine caregivers at Ola During Children’s Hospital and three caregivers at Makeni government Hospital were excluded from the survey because they did not consent to participate.

### Ethical approval

We sought ethical approval for this study from the Sierra Leone Ethics and Scientific Review Committee (SLESRC); Directorate of Policy and Planning, Ministry of Health and Sanitation (MoHS) and the study was approved on 1st September 2016 by SLESRC. Study nurses were trained to use the questionnaire and to behave in an ethical manner that allows for the appropriate conduct of the study. The nurses informed the caregivers/guardians about the purpose of the survey and that they had the right to participate or refuse participation in the study. They were also informed that appropriate treatment would be provided regardless of their refusal to take part in the study. Each caregiver was asked to sign a consent form to indicate their willingness to participate in the study. All data collected were also coded to prevent disclosure of the information to any third party.

### Outcome measures and data collection

We used a validated WHO quality of life questionnaire (WHOQOL-BREF questionnaire) that has been tested in resource-limited countries [18, 19]. The questionnaire consisted of three parts, and that include details of the caregiver, caregiver adherence estimates and quality of life questions. Interviewer-administered questionnaire format was used to collect data from caregivers. The Likert scale used in the WHOQOL-BREF questionnaire can be seen in Table 1 below.

**Table 1:** Five points Likert scale to measure the quality of life

Not at all or very dissatisfied or very poor or Never	A little or dissatisfied or poor or seldom	Moderately or neither satisfied nor dissatisfied or neither poor nor good or Quite often	Mostly or satisfied or good or well or very much or Well or very often	Completely or very satisfied or very good or very well or extremely or Always

The "Social domain sex life question was not used for the adaptation and utilisation of the WHOQOL-BREF paediatric age group questionnaire in this study because all of these children are aged 14 years and under and questions are being responded to, by their caregivers.

The dependent variables in this study were the four domains (Physical Health, Psychological Health, Environmental Health and Social Health) of the WHOQOL-BREF [18, 19].

The independent variables were the demographic variables adapted from a previous study that measures adherence in paediatric patients [20]. Adherence was assessed using self-reporting measures for adherence to Highly Active Antiretroviral Therapy (HAART) by caregivers, as shown in Table 2 below.

**Table 2:** Measures of Adherence

Non-adherence:	Any indication of missed dose in the past week/month, or a dose more than an hour late.
Adherence:	no indication of a missed dose

Such measures of adherence are still widely used in resource-limited countries [21]. However, other studies showed that caregiver reports could overestimate the level of adherence in paediatric HIV/AIDS patients [4, 20].

Four trained data collectors (two nurses working in each of the two HIV/AIDS clinics at Ola During Children’s hospital and Makeni Government hospital) collected the data through interviewer-administered format.

### Statistical analysis

Statistical package for social sciences (SPSS version 16.0) was employed during data analysis. Reliability and validity of the instrument were done by determining Cronbach’s alpha value for which an alpha value greater than or equals to 0.7 was deemed acceptable [22, 23] while correlations above 0.4 were considered to be acceptable [23]. Descriptive statistics were used to analyse categorical and continuous variables. Pearson’s correlation was used to determine the level of agreement between the two overall Quality of life questions and the four domains of the WHOQOL-BREF. Chi-square and Fisher’s exact tests were used to assess the association between the independent variables and the level of adherence (dependent variable) of paediatric patients to HAART. An independent t-test and analysis of variance tests were used to determine the association between participants’ characteristics and the average quality of life scores (transformed scores of four domains). Post hoc analysis was further conducted for domains that showed significant difference with caregivers’ or patients’ characteristics. Backward multivariate linear regression was employed to investigate the relationship between quality of life and patient characteristic with a *P*-value less than 0.05 considered statistically significant. For stepwise multivariate linear regression analysis, caregiver HIV status (positive, negative and Don’t Know) was grouped into binary data as positive and non-positive. The non-positive data includes the data for a patient with negative HIV status and patient with no knowledge of their HIV status. Relationship of caregivers (1-mother/Father/Sibling, 2-cousin/Aunties/uncles, 3-Neighbours/Relatives outside the home) was analysed as Nuclear family (1) and Extended family (2, 3) to determine the influence of close relatives against other family members on the quality of life of children with HIV/AIDS. The independent variable (adherence versus non-adherence) was also analysed to determine the influence on the dependent variable (quality of life of children with HIV/AIDS) using the backward linear regression analysis.

## Results

### Demographic and other related characteristics

Out of the 200 caregivers that were invited, 188 consented to participate, and their data were included in our final analysis. Table 3 shows that 74.5% of caregivers were aged 30 years or older, 76.6% of caregivers were female, 43.6% were HIV positive, and 33% of caregivers do not know their HIV status. Also, 60.6% of caregivers are members of the nuclear family. 62.8% of the caregivers had a problem with keeping to the timing of medication with 35.1% of this occurring in the morning, and 15.4% of problems occurring in the evening. More than half (56.9%) of the caregivers had difficulty in getting their child to take their medication. Close to two-thirds (61.7%) of children in this study were less than 5 years, 54.3% of the children were male, and 76.1% of the children were involved in an institutional nutritional program. Only 5.9% paediatric age group were adherent to Highly Active Antiretroviral Therapy (HAART) while 94.1% were non-adherent.

**Table 3:** Demographic and other Related Characteristics

Caregiver Age	Frequency	Percentage
< 30 years	48	25.5
≥ 30 years	140	74.5
**Care giver Sex**		
Male	44	23.4
Female	144	76.6
**Caregiver HIV Status**		
Positive	82	43.6
Negative	44	23.4
Don’t Know	62	33.0
**Caregiver Relationship to Child**		
Mother/Father/Siblings	114	60.6
Close relatives (Grandparents/Aunties/Uncles)	70	37.2
Neighbour/Relative outside home	4	2.1
**Child’s Sex**		
Male	102	54.3
Female	86	45.7
**Child’s Age**		
< 5 years	116	61.7
≥ 5 years	72	38.3
**Involved in Nutritional Program**		
Yes	143	76.1
No	45	23.9
**Problem with keeping to time of medication**		
Yes	118	62.8
No	70	37.2
**When does medicine administration problem occur?**		
Mornings	66	35.1
Evenings	29	15.4
Weekends	9	4.8
Weekdays	14	7.5
Not Applicable	70	37.2
**Problems in getting child to take medication**		
Yes	107	56.9
No	81	43.1
**Child’s HAART Adherence status**		
Adherent	11	5.9
Non-Adherent	177	94.1

### Factors affecting non-adherence

The study showed that three factors influenced paediatric HIV patient adherence to HAART, and they include child-related factors, caregiver related factors and institutional factors. Formulation problem (72.3%) and bitter medication (52.7%) were the most common child-related factors affecting paediatric HIV patient adherence to HAART. The commonest caregiver related factors were “didn’t want others to see” (61.7%), “was away from home” (60.6%), “didn’t have money to take child to the hospital” (56.9%), “forgetful” (38.8%) and “don’t know how to use the medication” (11.2%). In the case of institutional factor, 2.7% stated that “medicine was not available in the clinic”. (Table 4).

**Table 4:** Factors affecting adherence to highly active antiretroviral therapy

Factors	Yes	No
	N (%)	N (%)
**Child Related factors**		
Problem with formulation	52 (72.3)	138 (27.7)
Bitter medication	99 (52.7)	89 (47.3)
**Caregiver Related factors**		
I was away from Home	114 (60.6)	74 (39.4)
Always around child	7 (3.7)	181 (96.3)
Did not want others to see	116 (61.7)	72 (38.3)
Don’t Know how to use medicine	21 (11.2)	167 (88.8)
Too busy and forget	73 (38.8)	115 (61.2)
No money to take child to clinic	107 (56.9)	NA
**Institutional related Factors**		
Medicine was not available in the clinic	5 (2.7)	NA

*NA* Not Applicable, means answer was not provided by respondent

### Factors affecting adherence

No Statistical significant association was seen between independent variables and adherence to HAART (dependent variable) (Table 5).

**Table 5:** Association between independent variables and adherence to HAART

Characteristic	Chi-Square value	P-Value
Caregiver Grouped Age	1.661	0.197
Caregiver Sex	1.335	0.248
Caregiver HIV/AIDS status	4.347	0.093
Caregiver Relationship	0.250	1.000
Child's nutritional status	0.071	0.789
Child's age	3.174	0.075
Child's sex	< 0.010	0.984

### Assessment of quality of life in paediatric HIV/AIDs patient on HAART

Table 6 shows the average transformed scores of the four different domains with the psychological domain [63.1(SD 17.7] and the social relationship domain [44.1(SD 18.2)] being the highest and lowest mean scores respectively.

**Table 6:** Transformed Quality of Life (QOL) Domain Scores (*N* = 188)

QOL Domains	Minimum	Maximum	Mean (SD)
Physical Health	21.4	82.1	56.6 (11.6)
Psychological domain	4.2	91.7	63.1 (17.7)
Social Relationship	12.5	87.5	44.1 (18.2)
Environment	21.9	87.5	57.0 (13.6)

### Association of independent variables and quality of life domains

Table 7 shows that there is a statistically significant difference between caregiver HIV/AIDS status and physical health (*p* = 0.024) and psychological health (*p* <  0.001) domains. Also, a significant difference was observed between caregiver type and all the quality of life domains except social health. In addition, there was a statistically significant difference between adherence to HAART and the physical health domain. Participants who were adherent to HAART were more likely to have improved physical health and Significant difference in social health was also seen for caregivers accompanying children aged less than 5 years.

**Table 7:** Bivariate associations between independent variables and quality of life

Characteristics	Quality of Life Scores			
	Dom 1 Mean (SD)	Dom 2 Mean (SD)	Dom 3 Mean (SD)	Dom 4 Mean (SD)
**Caregiver Age**				
< 30 years	56.3 (11.0)	63.9 (15.4)	43.5 (21.9)	58.98 (12.2)
≥ 30 years	56.7 (11.9)	62.8 (18.4)	44.4 (16.9)	56.3 (14.1)
P-value	0.834	0.713	0.772	0.243
**Caregiver Sex**				
Male	56.1 (10.5)	64.9 (14.4)	40.3 (11.7)	58.1 (13.4)
Female	56.8 (12.0)	62.5 (18.6)	45.3 (19.6)	56.7 (13.7)
P-value	0.765	0.444	0.112	0.543
**Caregiver HIV status**				
Positive	58.3 (11.7)	68.2 (14.7)	42.5 (20.2)	59.3 (13.7)
Negative	58.2 (9.5)	61.5 (11.3)	47.2 (16.1)	53.8 (9.7)
Don’t Know	53.3 (12.4)	57.5 (22.6)	44.2((16.6)	56.2 (15.4)
P-value	0.024^a^	< 0.001^b^	0.397	0.081
**Involvement in Nutritional program**				
Yes	57.0 (11.5)	63.1 (16.9)	44.3 (17.3)	56.2 (12.5)
No	55.3 (12.2)	62.9 (20.2)	43.6 (20.9)	59.5 (16.7)
P-value	0.387	0.929	0.821	0.156
**Caregiver type (taking child to clinic)**				
Parents (Mother/Father/Siblings)	58.0 (10.6)	68.2 (14.8)	43.0 (18.2)	59.7(13.5)
Close relatives (Grandparents /Aunties/Uncles)	55.7 (11.6)	56.9 (17.1)	45.4 (18.2)	53.8 (12.3)
Neighbour/Relative outside home	34.8 (19.6)	26.0 (27.1)	56.3 (12.5)	36.7 (13.1)
P-value	< 0.001^b^	< 0.001^b^	0.280	< 0.001^b^
**Child’s Age**				
< 5 years	56.7 (10.8)	63.1 (16.5)	47.0 (18.7)	57.8 (13.9)
≥ 5 years	56.5 (12.9)	63.0 (19.5)	39.6 (16.4)	55.7 (13.1)
P-value	0.903	0.973	0.006^a^	0.312
**Child’s Sex**				
Male	55.2 (12.5)	62.0 (19.2)	44.7 (19.9)	57.0 (15.1)
Female	58.3 (10.3)	64.3 (15.8)	43.5 (16.0)	57.0 (11.7)
P-value	0.63	0.369	0.634	0.989
**Adherence status**				
Adherent	64.6 (8.1)	70.8 (13.0)	47.7 (24.3)	55.7 (12.0)
Non-Adherent	56.1 (11.7)	62.6 (17.9)	43.9 (17.8)	57.1 (13.6)
P-value	0.019^a^	0.134	0.502	0.742

^a^Significant; ^b^Very significant; *SD* standard deviation; Domain 1(Dom1): Physical Health; Domain 2(Dom2): Psychological Health; Domain 3(Dom3): Social Health Domain 4(Dom4): Environmental Health

Multivariate backward linear regression model after adjusting for other covariates revealed significant association in physical health domain with HIV status of caregiver and adherence status. Our study also revealed a significant association between caregiver type to psychological health and the environmental health domain. (Table 8).

**Table 8:** Backward multiple linear regression analyses of factors significantly associated with quality of life of paediatric HIV/AIDs patient on HAART

Characteristics	Quality of Life Scores			
	Dom1 Coef/***P***-value (95%CI)	Dom2 Coef/P-value (95%CI)	Dom3 Coef/P-Value (95%CI)	Dom4 Coef/P-value (95%CI)
**Caregiver HIV status**				
Positive Vs non-positive	−4.299/0.014			
	(−7.714, −0.883)			
**Caregiver type (taking**				
**Child to clinic)**				
Nuclear vs Extended Family		−11.458/< 0.001		−6.875/0.001
		(−16.653,-6.263)		(−10.775,-2.974)
**Adherence status**				
Adherent vs	−10.218/0.005			
Non-adherence	(−17.295,-3.141)			

Coef: Unstandardized Beta value; Domain 1(Dom1): Physical Health; Domain 2(Dom2): Psychological Health; Domain 3 (Dom3): Social Health Domain 4 (Dom4): Environmental Health

### Validity and reliability of questionnaire

Pearson’s Correlation was found to be significant for the four domains (Table 9) and Cronbach’s alpha was found to be 0.769.

**Table 9:** Correlation coefficients (CC) in two quality of life questions and four domains of the WHOQOL-BREF

		Q1	Q2	Dom1	Dom2	Dom3	Dom4
**Q1**	CC	1	0.750	0.626	0.719	0.221	0.407
	P-value		< 0.001	< 0.001	< 0.001	0.002	< 0.001
**Q2**	CC		1	0.727	0.794	0.29	0.455
	P-value			< 0.001	< 0.001	< 0.001	< 0.001
**Dom1**	CC			1	0.806	0.323	0.551
	P-value				< 0.001	< 0.001	< 0.001
**Dom2**	CC				1	0.335	0.628
	P-value					< 0.001	< 0.001
**Dom3**	CC					1	0.238
	P-value						0.001
**Dom4**	CC						1

CC: Correlation Coefficient; significant P-value at less than 0.05;

Q1: How can you rate your child’s quality of Life

Q2: How satisfied are you with your child’s health

Domain 1(Dom1): Physical Health; Domain 2(Dom2): Psychological Health;

Domain 3(Dom3): Social Health Domain 4(Dom4): Environmental Health

## Discussion

This study provides an empirical evidence on the level of adherence and quality of life as well as their associated determinants among children living with HIV/AIDS in Sierra Leone.

### Factors affecting non-adherence

The results of this study indicate that non-adherence among HIV positive paediatric patients was rife. Most caregivers had problems administering medication to their children in the morning than at any other time of the day. Such difficulties may be due to caregivers leaving home early in the morning for work/trade when the child is asleep or decided to skip dose due to the absence of food [24]. The key reasons for the high prevalence of non-adherence in our study were formulation related factors such as the taste of the medication. Caregiver related factors were fear of discrimination from others, lack of support and or fear of disclosure. Institutional factors were an absence of money to take the child to the hospital, inadequate knowledge on the use of medication and the shortage of HIV/AIDS drugs in the clinic. Previous studies had identified these factors affecting adherence [25, 26]. Non-adherence among children with HIV/AIDS means not achieving the high level of adherence of 95% or more. Non-adherence might result in sub-therapeutic blood concentrations, treatment failure, and the emergence of drug resistance with the resulting burden on the health system due to lengthy hospital stay and increased healthcare cost.

Therefore, the healthcare team must be involved in medication counselling for the paediatric age group. This would possibly require a separate counselling session that is focused on assessing adherence of paediatric patient/caregiver, providing information on the use of medication, possible HAART side effects and contraindication aimed at improving the factors affecting patient adherence to their medication.

There is also the need for the development of health policies or guidelines in all hospitals that take these factors (patient, institutional, caregiver) into consideration. Family support and community sensitisation and awareness are also crucial in preventing the stigma that may be associated with HIV/AIDS in the society.

Further assessment of demographic characteristic in our study showed that active involvement of nuclear family member (Father/Mother/Sibling) presented statistical significant difference in quality of life of paediatric patients (psychological health and environmental health domain). Other studies showed that involvement of a member of the nuclear family especially the mother or father [20] could improve adherence to HAART and caregiver report does correlate very well with viral load and clinical outcomes [27]. HIV/AIDS programs and community sensitisation interventions should encourage nuclear family members (if they are alive and well) towards active involvement in caring for their paediatric HIVAIDs patients. Studies in tropical regions showed that HIV/AIDs disclosure can improve adherence in children on HAART [28, 29]. Other studies also confirmed that disclosure of HIV status is a major issue for caregivers [30, 31].

### Assessment of quality of life in paediatric HIV/AIDs patient on HAART

Quality of life was assessed using descriptive data that collected information on means data variability inferential statistics of quality of life domains. The highest mean score was obtained in the psychological domain reflecting caregivers’ assessment of child’s happiness, acceptance of child’s bodily appearance and child’s negative feelings. The lowest score was seen in the social domain reflecting caregivers’ expressed dissatisfaction from friends and lack of support from other people with high variability in psychological and social domain compared to physical health and environmental health domain. The lowest mean score in the social domain of this study is similar to a study conducted in South India [31], Thailand [32] and China [33]. This shows the need for continual general public sensitisation, caregiver education on the positive effect of treatment compliance and the need for paediatric treatment prioritisation.

### Association of Independent variables and quality of life domains

A Post-Hoc analysis of HIV status (Positive, Negative and Don’t Know) of the quality of life domains revealed that the difference was more significant between the Positive and Don’t Know for Physical Health and Psychological Health and between the Positive and Negative for Environmental Health domain. Higher mean scores were observed for quality of life domains among children accompanied by HIV positive caregivers compared to children accompanied by caregivers that don’t know their status. Higher mean scores were also observed for children that were accompanied by HIV positive caregivers in the environmental health domain than children accompanied by HIV negative caregivers. Probably, HIV positive caregivers were actively involved in sensitisation, and counselling sessions organised by HIV program, seek information to improve their health status, are possibly on antiretroviral treatment themselves and so they can appreciate the need for the use of HAART in the suppression of viral load of the virus to enhance immunity [34].

Post- Hoc Analysis for caregiver type involved in paediatric HIVAIDs care revealed significant differences between the participation of nuclear family and extended family in the psychological and environmental domains. A significant difference was observed between the nuclear family and the other family type with a higher mean for nuclear family involvement than the involvement of extended family and other family types. This can be translated into better health outcomes when a member of the nuclear family is actively involved in caring for the child. The child’s physical health, psychological health and environmental health improved when an extended family member was involved in caring for the child than when other family type other than the nuclear family was involved. This provides an option for the influence of caregiver type on child’s health in the absence (or death) of a nuclear family member.

In this study, a backward multiple linear regression model was used after adjusting for other covariates to assess the independent variables that showed significant association with the dependent variables, revealing the associations below. The significant association between HIV status and physical health shows that a child accompanied by a caregiver without knowledge of HIV status had poorer Physical health than those accompanying caregivers with knowledge of their HIV status (positive/negative). Similarly, the results also showed that non-adherence was strongly correlated with poor physical health. The results of this study are further emphasised by UNAIDS best practices [34] and other studies on adherence which indicate a positive relationship between awareness and good health [28]. It is therefore important to ensure that caregivers of children with HIV/AIDS have knowledge of their HIV status because of its positive impact on the quality of life and the child’s adherence. This should aim at providing information about the influence of the knowledge of HIV status among caregivers and the possibility for improved health of paediatric patient on HAART. HIVAIDS program should use an opt-out testing for caregivers of paediatric patients.

Counselling sessions must focus on the reason for the use of HIV medications and their benefit in improving the quality of life of the paediatric age group. Caregivers must be informed about the high level of adherence required for the achievement of better physical health and good quality of life of paediatric HIV/AIDS patients.

The results of this study revealed that a child’s poor psychological health was more associated with whether the caregiver is from the extended family, as seen in a study in Kenya [35]. A study on paediatric HIV disclosure did not find statistically significant differences between pre-disclosure and post-disclosure quality of life [36]. Therefore, disclosure to child should be encouraged at an appropriate time. Another study in Kenya revealed a low prevalence of disclosure of HIV status to children with highlights of how disclosure may be related to key outcomes such as medication adherence, experiences of stigma and symptoms of depression [37].

The study also showed that poor social health is more associated with children age group greater than or equal to 5 years. This may be because of the commencement of schooling amongst this age group. A Nigerian study showed that schooling could also account for the factors contributing to poor adherence amongst children [38].

The regression model did not show any association between social health and the independent variables. The results of this study revealed that children had poor environmental health when the caregiver was a member of the extended family compared to when a nuclear family was involved.

### Assessment of bias

The method of caregiver report to assess adherence is widely used in adherence studies in low resource settings despite its possibility of overestimating adherence measures [39]. Other methods that can be used to assess the level of adherence are pill count, biological markers, medication event monitoring tool and other measures like pharmacy refill. There is need for further studies with the use of other measures of adherence rather than self-reporting by caregivers in order to provide more reliable evidence of measures of adherence among paediatric HIV/AIDS patients on HAART in these hospitals.

### Strengths and limitations of the study

The study demonstrates good internal consistency for the WHOQOL-BREF. Cronbach’s alpha value for assessing the reliability was 0.769 for the four quality of life domain scores. The validity was assessed using Pearson’s Correlation Coefficient with statistically significant correlations found among two overall quality of life questions and the four domains, and most correlations greater than 0.4. Similar findings were reported in other studies with low social domain correlation values though significant [19, 22, 23] and in paediatric age group using the GHAC questionnaire that also uses the quality of life domains as in the WHOQOL-BREF [32]. The demographic data and adherence questionnaire had also been used in several other studies that measure the adherence and quality of life of paediatric HIV/AIDS patients [20, 32].

To date, no study had been conducted in Sierra Leone to determine the quality of life of paediatric HIV/AIDS patients on Highly Active Antiretroviral Therapy and its association to therapeutic adherence. This study is a cross-sectional study of caregivers that fulfilled the inclusion criteria during the study period in the two hospitals, with a good sample size of 188 relatives to the available data of 383 children [13] that are receiving antiretroviral therapy in Sierra Leone. The result of this study is conservative and may not be used to generalise the whole population because of the small sample size and or the convenience sampling method used. These limitations should be considered when interpreting the results of this study.

The study was able to detect a statistically significant association with other variables, which suggests that it had enough power to be able to detect their association with adherence and/or quality of life.

## Conclusion

The study revealed a high percentage of non-adherence among paediatric HIV/AIDS patients receiving Highly Active Antiretroviral Therapy. The study showed that knowledge of HIV status, the involvement of nuclear family and HAART adherence is key for the improvement of physical health, while the involvement of nuclear family as a caregiver is key for improvement of psychological and environmental health. Therefore, the involvement of a member of the nuclear family in the treatment of children with HIV/AIDS and caregivers’ knowledge of their HIV status can improve adherence to treatment and improve quality of life of children living with HIV/AIDS.

## Data Availability

The datasets informing the findings of our study are available from the corresponding author on reasonable request.

## Abbreviations

HAART: Highly active antiretroviral therapy
HIV/AIDS: Human immunodeficiency virus/Acquired immunodeficiency syndrome
WHOQOL-BREF: WHO quality of life summary questionnaire
UNAIDS: United Nations Joint programme on HIV/AIDS
PLHIV: People living with HIV
CLHIV: Children living with HIV
ODCH: Ola During Children’s Hospital
SLESRC: Sierra Leone Ethics and Scientific review committee
MoHS: Ministry of Health and Sanitation
SPSS: Statistical package for social sciences
